# Multidrug-resistant Gram-negative bacteria in lung transplantation: a narrative review of infectious complications and current management

**DOI:** 10.3389/fmicb.2025.1674976

**Published:** 2025-12-10

**Authors:** Margherita Sambo, Gabriele Giuliano, David Bennett, Luca Luzzi, Federico Franchi, Mario Tumbarello, Francesca Montagnani

**Affiliations:** 1 Department of Medical Biotechnologies, University of Siena, Siena, Italy; 2 Unit of Infectious and Tropical Diseases, Siena University Hospital, Siena, Italy; 3 Unit of Respiratory Diseases, Siena University Hospital, Siena, Italy; 4 Unit of Lung Transplant, Siena University Hospital, Siena, Italy; 5 Department of Medical Sciences, Surgery, and Neurosciences, University of Siena, Siena, Italy; 6 Unit of Cardiothoracic and Vascular Anesthesia and Intensive Care Unit, Siena University Hospital, Siena, Italy

**Keywords:** lung transplantation, multidrug resistant Gram-negative bacteria, difficult to treat infections, immunocompromised host, antibiotic prophylaxis

## Abstract

Multidrug-resistant Gram-negative bacterial (MDR-GNB) infections represent a major challenge in lung transplantation (LuTx), due to their possible association with poor clinical outcomes and the limited availability of effective antimicrobial agents. Nevertheless, MDR-GNB colonization or infection is no longer considered an absolute contraindication to transplantation. Recent recommendations issued by leading societies in clinical microbiology and infectious diseases, national expert groups, and transplant medicine professionals provide updated recommendations on antimicrobial strategies, encompassing both established and newly approved agents. Following an overview of the epidemiology of infections in LuTx recipients, with a focus on the specific impact of MDR-GNB, this review aims to explore current evidence on diagnostic approaches, prophylactic measures, and therapeutic management. Remaining knowledge gaps in this area are also highlighted, emphasizing the need for further research to optimize prevention and treatment strategies in this high-risk population.

## 1 Introduction

Lung transplantation (LuTx) is a treatment option for selected adult patients with end-stage chronic respiratory diseases. These include chronic obstructive pulmonary disease (COPD), with or without α1-antitrypsin deficiency, interstitial lung diseases, cystic fibrosis (CF), and idiopathic pulmonary arterial hypertension. During years, improved post-LuTx survival and advances in transplantation techniques have led some centers to also consider conditions such as sarcoidosis, non-cystic fibrosis bronchiectasis, lymphangioleiomyomatosis, and idiopathic pulmonary arterial hypertension as eligible for transplantation (Lund et al., 2017). In last years, transplantation has also to be considered as an option in case of acute lung injury: COVID-19 pandemics has resulted in a substantial number of patients progressing to acute respiratory failure, leading lung transplantation experts to offer this opportunity also to these patients. Yeung et al. (2021) demonstrated in the Toronto Lung Transplant Program experience, that LuTx resulted in a rescue opportunity also in acute lung injury. More other similar experiences were subsequently published: Chizinga et al. (2022) demonstrated that patients who underwent lung transplantation for acute exacerbations of interstitial lung disease had no meaningful difference in overall survival, rate of primary graft dysfunction or acute rejection compared with those transplanted with stable disease. Across different cohorts, the proportion of lung transplants performed for acute lung injury has been reported to range between 0.15 and 5.7% (Guidot et al., 2023; Harano et al., 2021).

Infectious complications and primary graft dysfunction after LuTx are major causes of early mortality and they are also associated with significant morbidity, including prolonged hospitalization, the need for mechanical ventilation or chronic supplemental oxygen, and intensive respiratory rehabilitation. According to some evidence, although not conclusive, these complications may also contribute to the development of chronic rejection (Lund et al., 2017).

Infections after LuTx, as far as in other solid organ transplants (SOT), follow a time-dependent pattern. Bacterial infections are most frequent during the first 30 days after transplantation. They remain clinically significant over the following 3–6 months, although their incidence gradually declines. Between the second and sixth month after transplantation, viral reactivations, particularly cytomegalovirus (CMV), and opportunistic infections such as invasive molds become more common. Infections occurring after 6 months are usually caused by community-acquired pathogens (Joean et al., 2022).

Among bacterial infections, pneumonia is the most frequent. However, other serious conditions can also occur, including deep surgical site infections (SSIs), empyema, wound infections, mediastinitis, sternal osteomyelitis, and pericarditis (McCort et al., 2021).

Epidemiological data show that Gram-negative bacteria (GNB) play a predominant role in the etiology of post-LuTx bacterial infections. This includes both Enterobacterales and non-fermenting Gram-negative bacteria (NFGNB).

The association between LuTx and GNB has been recognized for a long time. *Burkholderia cenocepacia* was the first notable pan-resistant organism identified as negatively impacting survival following LuTx. Pre-transplant colonization with this pathogen is considered a traditional relative contraindication to LuTx that can lead patient not suitable to transplant in some centers (Hardin, 2024).

With the global rise in antimicrobial resistance, for some time now, infections caused by MDR-GNB are increasingly affecting SOT recipients, including LuTx (Bartoletti et al., 2018).

In this context, several studies have investigated clinical outcomes in patients infected or colonized with MDR-GNB. Patients with pre-transplant colonization by pan-resistant *Pseudomonas aeruginosa* have demonstrated post-transplant survival comparable to other cystic fibrosis patients. However, data remain limited regarding the post-transplant impact of infections caused by *Achromobacter xylosoxidans* and *Stenotrophomonas maltophilia* (Hadjiliadis et al., 2007).

Fortunately, the development of new antimicrobial therapies and the advent of more rapid and accurate diagnostic tools have significantly improved the management of these infections. As a result, even patients previously excluded from transplantation due to colonization with multidrug-resistant (MDR) pathogens are now being considered for LuTx.

This narrative review aims to present the current understanding of the role of MDR-GNB in the epidemiology of post-LuTx infections and their impact on clinical outcomes; to describe diagnostic tools and algorithms for infection risk stratification and for the early identification of patients who are most likely to benefit from targeted therapy; and to summarize current therapeutic strategies and options tailored to specific MDR-GNB scenarios, while outlining existing knowledge gaps and identifying key research questions that warrant further investigation.

## 2 Epidemiology of bacterial infections in lung transplantation

In the early post-operative period, the lung allograft demonstrates a higher susceptibility to infections compared to other SOT (Fishman, 2017).

The respiratory tract is the most commonly affected site of infection following LuTx and bacterial pneumonia represents the most frequent infectious complication with the greatest impact on early mortality (Burguete et al., 2013).

Moreover, more recent studies confirm a concerning trend in the incidence of infections among lung transplant recipients. Wojarski et al. (2018) described a cohort of 97 lung transplant patients, 69% of whom developed pulmonary bacterial infections during hospitalization (Wojarski et al., 2018). Similarly, another cohort study reported a post-transplant bacterial infection rate of 81% (90/111) (Lv, 2024).

According to the Registry of the International Society for Heart and Lung Transplantation (ISHLT), infection is the leading cause of death within the first-year post-transplant, accounting for approximately 31% of early mortality cases (Christie et al., 2011). More recent data indicate a slight increase in mortality due to infections among lung transplant recipients (LTRs). According to a Chinese cohort study of 549 LTRs, the 1-year, 2-year, and 3-year mortality rates among patients who developed pulmonary bacterial infections within 30 days after transplantation reached 37.7, 45.2, and 49.9%, respectively (Gao et al., 2024). Infection rates can vary across centers. For instance, Han et al. (2025) reported an infection-related mortality rate of 20% (4/20) among patients undergoing primary lung transplantation, which increased to 70% (7/10) in the retransplantation group.

Notably, growing evidence suggests that infections not only contribute to direct mortality but may also play a pivotal role in the pathogenesis of bronchiolitis obliterans syndrome (BOS), may be due to an amplification of the inflammatory response, increasing donor-specific alloreactivity through the expression of HLA and producing deleterious cytokines (Parada et al., 2010).

### 2.1 Risk factors

Lung allografts are constantly exposed to microorganisms from both the external environment through inhalation and the bloodstream. As a result, LuTx recipients are particularly vulnerable to infectious complications. Moreover, surgical procedures involved in LuTx cause significant anatomical alterations that compromise the effectiveness of innate immunity and physical barriers.

Following LuTx, bronchial circulation is disrupted, and epithelial integrity is lost. Denervation of the allograft often suppresses the cough reflex, promoting bronchial hyperresponsiveness (Levine, 1999), while phrenic nerve damage plays a pivotal role in diaphragm function, causing longer intensive care unit (ICU) stays, increased reintubation rates, and more frequent use of NIV (Palleschi et al., 2024).

In addition, lymphatic drainage is impaired, resulting in stasis and edema of the bronchial tissues. This, combined with potential stenosis or necrosis at the bronchial anastomosis site, creates a favorable environment for microbial colonization and growth, while also impairing clearance of bronchial secretions (Murthy et al., 2010).

Furthermore, the use of nasogastric and endotracheal tubes increases the risk of aspiration and infection (Cook and Kollef, 1998). In fact, Walti et al. (2024) demonstrated how the duration of mechanical ventilation was associated with the development of pneumonia, predominantly VAP, early after lung transplantation in about 10% of LuTx recipients. They also recognized pre-transplant pulmonary hypertension and pre-transplant immunosuppression as risk factors for pneumonia occurrence.

Beyond anatomical factors, induction and maintenance immunosuppressive therapy—essential to prevent acute and chronic rejection—also increases infection risk by impairing cellular immunity.

Finally, colonization or infections present in either the recipient or in donor can further elevate the risk of post-transplant infectious complications (Bonvillain et al., 2007; Bunsow et al., 2020; McCort et al., 2021).

**Figure 1:**
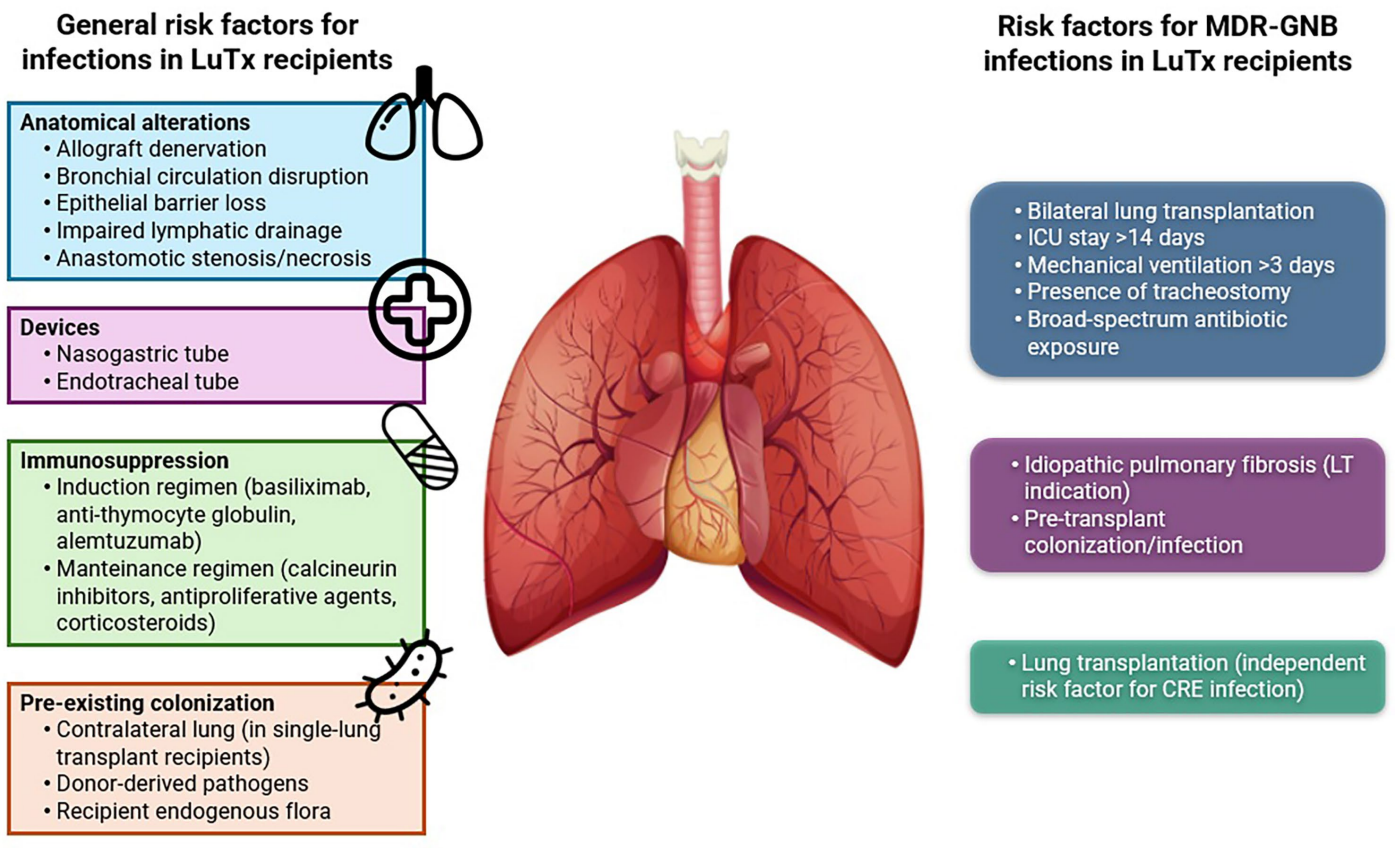
Risk factors for MDR-GNB infections in LuTx recipients.

Figure 1 (left) illustrates the principal risk factors contributing to infectious complications in LuTx.

### 2.2 Etiology

GNB are reported as the leading cause of bacterial infections in LuTx, accounting for nearly three-quarters of all cases, whereas Gram-positive bacteria are less frequently involved. This distribution was described in a Spanish prospective study, where *Pseudomonas aeruginosa* emerged as the most common pathogen, responsible for 43.4% of infections. It was followed by *Staphylococcus aureus* (34.9%), Enterobacterales (9.6%), and other NFGNB, including *Acinetobacter baumannii* and *Burkholderia cepacia* (Campos et al., 2008).

A similar etiological pattern was confirmed by Aguilar-Guisado et al. (2007) in the multicenter RESITRA cohort study, which prospectively investigated post-transplant pneumonia in LuTx recipients.

A recent retrospective study conducted in China on 549 LuTx recipients reported a postoperative pulmonary bacterial infection rate of 82.7%. The predominant pathogens isolated within the first 30 days after surgery were *A. baumannii* (29.6%), *Klebsiella pneumoniae* (16.2%), *P. aeruginosa* (15.4%), *S. maltophilia* (13.9%), and *S. aureus* (4.8%) (Gao et al., 2024). Although etiological patterns may vary over time and across geographical regions, these findings are in line with previous reports and may reflect an increasing prevalence of *A. baumannii* in more recent years.

When talking about MDR-GNB, there are different resistance mechanisms that should be taken into account.

Enterobacterales have developed resistance to antibiotics such as *β*-lactams mainly through the production of enzymes capable of hydrolyzing these molecules, known as *β*-lactamases.

According to the Ambler classification, β-lactamases are divided into classes A, C, and D, which are characterized by a serine residue in the active site, and class B, which includes metallo-β-lactamases that require a zinc ion for catalytic activity.

The principal β-lactamases include:

- ESBLs (Ambler class A)—Extended-spectrum *β*-lactamases (predominantly CTX-M family, e.g., CTX-M-15) hydrolyse third-generation cephalosporins and are widespread in *E. coli* and *Klebsiella* spp.; plasmid carriage enables rapid dissemination (Lau et al., 2024).
- AmpC *β*-lactamases—Chromosomal or plasmid-mediated AmpC enzymes confer resistance to many cephalosporins and are often not inhibited by classical β-lactamase inhibitors; co-production with ESBLs complicates phenotypic detection and therapy (Etemadi et al., 2020).
- Carbapenemases (Ambler classes A, B, D)—Major enzymes include class A KPC, class B metallo-β-lactamases (NDM, VIM, IMP) and class D OXA-48-like enzymes. These enzymes hydrolyse carbapenems (to varying degrees) and are frequently plasmid- or mobile-element-associated, driving the global spread of carbapenem-resistant Enterobacterales (CRE) (Carroll et al., 2024).

*Pseudomonas aeruginosa* can exhibit diverse resistance mechanisms, extending beyond β-lactamase production to include several additional adaptive pathways such as:

- Carbapenemases and acquired β-lactamases—*P. aeruginosa* can carry acquired carbapenemases including class A (e.g., GES, occasionally KPC) and class B metallo-β-lactamases (e.g., VIM, IMP, NDM). These acquired enzymes vary geographically (Tenover et al., 2022).
- Intrinsic mechanisms—*P. aeruginosa* can commonly become carbapenem-resistant via loss or downregulation of the OprD porin (reducing carbapenem entry), overexpression of chromosomal AmpC, and upregulation of efflux pumps (e.g., MexAB-OprM, MexXY), which together raise MICs across multiple antibiotic classes (Yoon and Jeong, 2021).

Finally, *Acinetobacter baumannii* can exhibit one of the most complex antimicrobial resistance systems among all Gram-negative bacteria, often co-expressing multiple mechanisms simultaneously (Marino et al., 2024). These include:

- OXA-type carbapenemases (Ambler class D) - OXA-type enzymes (e.g., OXA-23, OXA-24/40, OXA-58 and OXA-51-like intrinsic enzymes) are the most frequent carbapenemases in A. baumannii and are major drivers of carbapenem resistance worldwide.
- Efflux pumps and regulatory mutations—Overexpression of pumps (notably AdeABC) driven by mutations in two-component regulators (AdeRS) or insertion sequences increase resistance to many drug classes.
- Porin / permeability changes and combined mechanisms—Reduced porin expression / porin modifications and the accumulation of multiple mechanisms (OXA enzymes + efflux + porin loss) commonly produce high-level, often XDR phenotypes.

### 2.3 The burden of MDR-GNB in LuTx recipients

Bartoletti et al. (2018) reported a significant incidence of multidrug-resistant organisms (MDROs) in LuTx recipients, ranging from 31 to 57%.

An Italian prospective study investigated colonization and infection due to carbapenem-resistant Enterobacterales (CRE) in a cohort of liver and lung transplant recipients. They analyzed rectal swabs, cultures of urine and, only for lung recipients, of BAL. Among 113 first-time LuTx recipients, 3 (2.7%) were CRE-colonized prior to transplantation, while 16 additional recipients (14.4%) became positive during follow-up—primarily with *K. pneumoniae* carbapenemase-producing strains (KPC-Kp). Approximately one-third of these patients developed a KPC-Kp infection (Errico et al., 2019).

Although prevalence and incidence data of MDROs infections in this population can be difficult to quantify due to study heterogeneity, important insights into related mortality have been provided by Giannella et al. (2023). In particular, they analyzed these specific data described below.

In a recent study by Boscolo et al., 33 of 132 lung transplant recipients (25.0%) developed an ESBL producing Enterobacterales infection, mostly presenting as pneumonia, with an overall mortality of 27% among LTRs with MDR Gram negative bacterial infections (Boscolo et al., 2022).

The prevalence of CRE infections after solid organ transplantation (SOT) is particularly high (5–7%) among lung transplant recipients (LTRs) (Errico et al., 2019; Raviv et al., 2012). In a cohort of 136 LTRs, 9 patients (6.6%) developed a CRE infection, with a 1-year mortality rate of 63.7% (Raviv et al., 2012). Furthermore, in a cohort of 897 SOT recipients, including 58 LTRs, mortality was significantly higher in patients with CRE bloodstream infections (BSI) compared to those with non-CRE BSI (17% vs. 6%, *p* < 0.001); when adjusted for confounding variables, CRE infection emerges as an independent predictor of poor outcomes (Anesi et al., 2023).

Difficult-to-treat *Pseudomonas aeruginosa* is among the most frequently isolated pathogens in lung transplant recipients (LTRs), with multidrug-resistant rates ranging from 7 to 50% (Palacio et al., 2015; Stjärne Aspelund et al., 2018; Tebano et al., 2016).

As to concern carbapenem-resistant Acinetobacter baumannii (CRAB), a Chinese study including 107 lung transplant recipients found that CRAB was the predominant MDR pathogen, accounting for 35% (28/87) of all MDR Gram-negative bacteria isolated. To conclude, CRAB was associated with a 30-day mortality of 5.9%, increasing dramatically to 66.7% at 1 year post-transplant (Meng et al., 2022; Nunley et al., 2010).

Recognizing risk factors for MDR-GNB infections in LuTx recipients is essential to implement effective strategies aimed at reducing their incidence.

Among SOT procedures, LuTx itself has been identified as an independent risk factor for CRE infections, as demonstrated in a recent study by Freire et al. (2024).

Additional risk factors associated with MDR bacterial colonization during intensive care unit stay include bilateral transplantation, ICU stay longer than 14 days, mechanical ventilation for more than 3 days, presence of a tracheostomy, and extensive exposure to broad-spectrum antibiotics (Tebano et al., 2016).

Paglicci et al. (2021) also investigated specific risk factors for MDR-GNB infections in LuTx recipients, identifying idiopathic pulmonary fibrosis as the indication for LuTx and pre-transplant colonization or infection as variables significantly associated with increased risk.

Figure 1 (right) illustrates the principal risk factors contributing to MDR-GNB infections in LuTx recipients.

### 2.4 The impact of donor-derived infections on post-LuTx MDR-GNB events

Infections caused by carbapenem-resistant Gram-negative bacteria (CR-GNB), such as CRAB and CRE, have reached alarming incidence rates across Europe in recent decades (European Centre for Disease Prevention and Control, 2023).

In LuTx recipients, MDR-GNB infections may also be transmitted via the donor’s graft, resulting in donor-derived infections (DDIs). These infections are particularly challenging to diagnose, potentially leading to delayed treatment and high mortality. Preventing such complications requires accurate diagnosis and appropriate treatment of infections in the donor (Cervera et al., 2014).

Even if as a week recommendation, recent Spanish guidelines suggest, if logistically feasible, administering inhaled and systemic antibiotics to lung donors with MDR-GNB colonization prior to organ procurement can be beneficial (Rodríguez-Goncer et al., 2025).

Although CR-GNB infection in a potential donor is typically considered a contraindication to SOT transplantation (European Directorate for the Quality of Medicines and HealthCare, 2022), a second opinion is suggested in some setting to evaluate case by case eligibility of the donor and of the specific organ (National Transplantation Center, Istituto Superiore Sanità, Italy, 2024).

Again, recent Spanish recommendations suggest that donation from donors with MDR GNB infection should not contraindicated if active antibiotics are available, the donor has received 48 h of appropriate treatment, the infection source is under control, and the recipient receive targeted antibiotic prophylaxis (Rodríguez-Goncer et al., 2025).

Anyway, culture results are often unavailable at the time of organ procurement. Consequently, donor infection may only be diagnosed after transplantation, unexpectedly exposing the recipient to a DDI. If donor culture results are pending at the time of LuTx, the risk of post-transplant infection increases.

Despite some limitations, the findings of Zhou et al. (2024) suggest that real-time screening of donor lungs for CR-GNB may help reduce CR-GNB-related DDI mortality.

Risk factors for donor colonization or infection with MDROs include prior surgical procedures (especially abdominal), prolonged mechanical ventilation, extended ICU stay, and exposure to broad-spectrum antibiotics—all of which may contribute to the risk of DDI (Lewis and Sifri, 2016).

Once donor infection is identified, effective and timely communication of microbiological results to the recipient’s transplant team is essential. Lewis and Sifri (2016) emphasized that early administration of targeted pre-emptive antibiotic therapy in recipients of grafts from MDRO-colonized donors significantly reduces the risk of transmission. They also advised against disregarding transient bacteremia or perfusate contamination even in cases where blood cultures turn negative after the initiation of donor antibiotic therapy.

Screening of preservation fluid (PF) for pathogen colonization has been recommended to limit DDIs risk (Cervera et al., 2014).

In SOT recipients whose PF tested positive for high-risk organisms, targeted prophylaxis has shown protective effects (Oriol et al., 2019).

Conversely, when PF is positive for CR-GNB, severe DDI-related complications have been reported (Giani et al., 2014).

To this end, Mularoni et al. (2024) recommend routine PF culture at the transplantation center. Detection of CR-GNB in PF should prompt heightened microbiological surveillance, including drainage culture (if present), enhanced monitoring, and targeted antimicrobial prophylaxis. They emphasize that rapid identification of high-risk DDIs recipients—regardless of the pathogen’s resistance profile—may be crucial to optimizing clinical outcomes (Mularoni et al., 2024).

Interestingly, a recent retrospective single-center study revealed that, regardless of DDIs occurrence, the presence of a positive bronchoalveolar lavage (BAL) in the graft was associated with early graft dysfunction, longer mechanical ventilation, and extended ICU stay—but only in previously uncolonized recipients. In contrast, pre-colonized recipients showed better early ICU outcomes, despite the risk of post-LuTx recolonization, maybe due to an underlying immunological tolerance. These findings suggest that the impact of any BAL positivity of the graft on early outcomes may differ based on the recipient’s colonization status prior to transplant (Fumagalli et al., 2024).

## 3 Diagnostics

### 3.1 Active surveillance

Even though risk factors for CRE infections are most frequently described in liver transplantation due to abdominal surgery, a significant correlation was demonstrated in heart transplantation, mainly linked to surgical complications and reflecting the strong association between MDR-GNB infections and healthcare-related settings (Taimur et al., 2021).

In SOT other than LuTx, both pre- and post-transplant MDR-GNB carriage and perioperative complications have been associated with an increased risk of developing CRE infections. Notably, a shorter interval between pre-transplant detection of CRE carriage and the procedure itself has been correlated with a higher risk of subsequent infection (Giannella et al., 2019; Taimur et al., 2021).

Active surveillance—including regular screening of respiratory specimens (e.g., sputum, tracheal aspirates, bronchoalveolar lavage) and rectal, cutaneous or nasopharyngeal swabs in both donors and recipients—plays a critical role in managing the perioperative period. Microbiological knowledge of the donor can guide perioperative prophylaxis and empirical treatment if early post-transplant infections occur. Likewise, awareness of recipient colonization status allows more accurate management of early complications after LuTx.

Indeed, the risk of post-transplant infectious complications should be stratified based on pre-transplant microbiological findings, along with indication for LuTx, type of procedure, and comorbidities (Paglicci et al., 2021).

Supporting this approach, Errico et al. (2019) conducted a prospective cohort study including liver and lung transplant recipients, showing that 14.4% of lung recipients acquired CRE colonization during hospitalization, with CRE infections occurring in 5.3% of cases—mostly among previously colonized patients. These findings underscore the extensive circulation of CRE and the risk of cross-contamination in transplant centers.

Certain LuTx indications, particularly cystic fibrosis (CF), pose unique challenges. In CF patients, *P. aeruginosa* is the most isolated organism, often co-existing with MDR-GNB such as *Escherichia coli* and *K. pneumoniae*. These pathogens tend to chronically colonize the airways and frequently cause post-transplant infections requiring targeted therapy (Congedi et al., 2023).

A well-documented microbiological history is thus essential to develop a tailored therapeutic strategy for infectious complications in these patients.

Furthermore, new-onset colonization by GNB is associated with other non-infectious complications. Botha et al. (2008) demonstrated that *P. aeruginosa* colonization of lower respiratory tract is significantly associated with the development of BOS. Interestingly, persistent colonization—likely originating from upper airway reservoirs in CF—does not appear to increase the risk of BOS, possibly due to immunological tolerance. In contrast, *de novo* colonization of the allograft seems to act as a marker of early airway damage and a precursor to BOS. Similar conclusions have been reported by Orfanos et al. (2018), who found that de novo GNB colonization after transplant is associated with an increased risk of chronic lung allograft dysfunction (CLAD) and reduced CLAD-free survival, compared to recolonization in previously colonized recipients.

These findings emphasize the importance of assessing both pre-existing and newly acquired colonization—mainly respiratory and rectal—to optimize the management of infectious complications. Moreover, the impact of early post-operative colonization on outcomes highlights the need for continued microbiological surveillance not only before but also after transplantation, particularly throughout the hospital stay.

### 3.2 Diagnostic approach to MDR-GNB infections in LuTx recipients

To optimize the management of bacterial infections in transplant recipients, obtaining an etiological diagnosis is crucial—particularly when MDROs are suspected. In these cases, collecting multiple clinical specimens for culture is mandatory. This approach not only allows for antimicrobial susceptibility testing but also provides valuable epidemiological and molecular information.

Regardless of resistance mechanisms, bacteria commonly responsible for infections in SOT recipients generally grow on standard culture media at 37 °C in aerobic conditions. The identification of MDR phenotypes is based on susceptibility data obtained through standardized methods such as disk diffusion, antibiotic gradient strips (e.g., E-test), agar dilution, and broth microdilution—often performed via automated systems. These techniques enable the determination of minimum inhibitory concentrations (MICs). Interpretation is typically based on breakpoints established by the Clinical and Laboratory Standards Institute (CLSI) and the European Committee on Antimicrobial Susceptibility Testing (EUCAST).

To improve efficacy and curb resistance development, synergistic combination therapy may lower MICs below resistance thresholds. Therefore, synergy testing—such as E-test-based synergy or time-kill assays—should be readily available in transplantation centers to guide personalized treatment strategies.

Given the vulnerability of immunocompromised hosts, time-to-treatment is critical. In this context, molecular diagnostic tools have become an invaluable resource.

One of the most impactful innovations in recent years is the application of proteomics-based techniques. Among these, matrix-assisted laser desorption/ionization time-of-flight (MALDI-TOF) mass spectrometry has transformed microbiological diagnostics. This method enables rapid identification of a broad spectrum of pathogens, including MDR isolates, within minutes, greatly outperforming conventional phenotypic approaches in terms of speed (Cervera et al., 2014).

Another important advance is the use of polymerase chain reaction (PCR)-based syndromic panels, which enable simultaneous detection of multiple pathogens from a single sample. These panels support early diagnosis, appropriate antimicrobial prescription, and prompt intervention. Typically organized by clinical syndrome (e.g., respiratory, gastrointestinal, neurological), they can also detect bloodstream pathogens and associated resistance genes, reducing both morbidity and mortality (Cassidy et al., 2021).

However, despite their speed and sensitivity, syndromic panels are not without limitations. High sensitivity may lead to detection of colonizing or non-pathogenic organisms, complicating clinical interpretation. Overdiagnosis can result in overtreatment or unnecessary laboratory testing. Hence, proper integration into laboratory workflows and ongoing validation are essential (Ramanan et al., 2018).

Lombardi et al. (2025c) emphasized the potential of syndromic panels in immunocompromised patients. They advocate their use alongside culture methods, particularly in analyzing donor BAL samples pre-transplant. This approach could rapidly identify pathogens not covered by prophylactic regimens, especially in front of MDR pathogens with resistance mechanisms detected by rapid molecular diagnostics, and help assess microbial clearance in the early post-transplant phase, potentially supporting early de-escalation of antibiotics. In the first case, as mentioned above, this method could permit the quick optimization of antibiotic therapy in the first fundamental phases of lung transplantation. On the contrary, if neither resistance mechanisms nor donor derived infections are recognized, an early antibiotic de-escalation should be recommended in order to preserve the patient’s microbiome.

In conclusion, despite their differences in speed, sensitivity, specificity, and accuracy, all diagnostic methods contribute uniquely and complementarily to the comprehensive management of MDR-GNB infections in LuTx recipients.

## 4 Antimicrobial treatment

### 4.1 Peri-operative antimicrobial prophylaxis

SSIs caused by MDR-GNB are on the rise following various surgical procedures, often preceded by rectal colonization (Righi et al., 2023b; Souverein et al., 2019).

Given that decolonization of MDR-GNB carriers before surgery is not routinely recommended due to its limited long-term efficacy and the potential for selecting further resistance, perioperative antimicrobial prophylaxis (PAP) remains a crucial strategy to prevent postoperative infections, particularly in CRE carriers undergoing surgical procedures, including SOT (Tacconelli et al., 2019).

However, the limited evidence in this field poses challenges to reaching a strong consensus among experts, leaving PAP strategies in SOT undefined. In 2020, Coiffard et al. (Coiffard et al., 2020) conducted a global survey on perioperative surgical prophylaxis in LuTx and found significant variability in practice. Despite existing clinical guidelines recommending cefazolin for antimicrobial prophylaxis in heart and lung transplantation (Anesi et al., 2018), over 70% of transplant centers reported using additional coverage targeting GNB and a great variability among centers has been reported (Lombardi et al., 2025a).

This may reflect both clinical experience and widespread documentation of GNB in lung transplant recipients.

Pretransplant respiratory bacterial colonization has been recognized as a key predictor of postoperative infection risk (Bonvillain et al., 2007). In support of this, the aforementioned surveys reported that most of prescribers tailored PAP based on pretransplant MDR-GNB sputum colonization (Coiffard et al., 2020; Lombardi et al., 2025a).

As for examples, in recipients with no prior colonization, piperacillin/tazobactam was the most commonly used anti–Gram-negative antibiotic among the participating centers (32.3%), followed by fourth-generation cephalosporins such as cefepime (21.2%). Approximately one-third of centers administered prophylaxis for 7 days (33.3%). In cases of known colonization by MDR *P. aeruginosa*, postoperative antimicrobial prophylaxis most commonly included meropenem or imipenem (92.9%), tobramycin (45.5%), and colistin (36.3%). A combination of a carbapenem (or a new antipseudomonal cephalosporin with a β-lactamase inhibitor) plus tobramycin or colistin was recommended in 69.7% of centers. In these cases, the duration of antibiotic treatment was at least 14 days in 66.7% of centers (Coiffard et al., 2020).

Recently, the European Society of Clinical Microbiology and Infectious Diseases with the European Committee on Infection Control (ESCMID/EUCIC) released updated clinical guidelines for PAP in patients colonized by MDR-GNB, providing stratified recommendations based on specific pathogens. Active surveillance for rectal colonization with MDR-GNB is advised for all pathogens, although the strength of recommendations varies. Notably, a good practice statement—though ungraded—supports the use of targeted PAP for all SOT recipients colonized by extended-spectrum cephalosporin-resistant Enterobacterales (ESCR-E), recommending agents based on susceptibility testing and advising to avoid carbapenems whenever possible. Conversely, due to insufficient evidence, the guidelines refrain from recommending PAP for recipients colonized by other MDR-GNB such as CRE or CRAB (Righi et al., 2023a).

The Spanish Society of Transplantation (SET), in collaboration with the Transplant Infection Study Group (GESITRA) of the Spanish Society of Infectious Diseases and Clinical Microbiology (SEIMC) and the Spanish Network for Research in Infectious Diseases (REIPI), released guidelines in 2018 for managing MDR-GNB infections in SOT recipients (Aguado et al., 2018) which have been recently updated in 2025 (Rodríguez-Goncer et al., 2025).

While their recommendations for ESCR-E align with ESCMID/EUCIC (albeit using a different grading system), discrepancies arise with other pathogens. In 2018 published version, for CRE-colonized donors or recipients, standard surgical prophylaxis was advised, except in centers with a high incidence of CRE-related surgical site infections, where tailored regimens may have been considered. In cases of MDR *P. aeruginosa* colonization, standard prophylaxis was advised only for non-lung transplants, while targeted PAP was recommended for LuTx recipients with cystic fibrosis or bronchiectasis and underlying infectious lung disease. In 2025 update, they finally suggest that in lung transplant recipients standard surgical prophylaxis should be adjusted if there is evidence of donor MDR GNB colonization or infection in respiratory samples (Aguado et al., 2018; Rodríguez-Goncer et al., 2025).

Finally, based on evidence showing a high rate of colistin-induced nephrotoxicity and its ineffectiveness in preventing surgical site infections when used as prophylaxis, and considering that prior exposure to carbapenems and colistin is a major risk factor for the development of resistance to these antibiotics, Spanish guidelines recommend that patients colonized with *A. baumannii* receive a standard prophylactic regimen targeting common skin pathogens, thus excluding the use of both carbapenems and colistin (Dijkshoorn et al., 2007; Freire et al., 2014; Qureshi et al., 2015).

The American Society of Transplantation (AST) takes a type-specific approach, recommending broader-spectrum prophylaxis in LuTx and in cases of delayed chest closure. If the donor or recipient has a history of pretransplant pulmonary colonization/infection, a weak recommendation is made to provide prophylaxis covering those organisms, especially in MDR-GNB endemic regions (Abbo et al., 2019).

In contrast, stronger recommendations exist regarding the duration of PAP for non-renal SOT, extending PAP to 48–72 h may be considered based on transplant type (Righi et al., 2023a).

Still, most centers tend to limit PAP duration if donor and recipient cultures are negative (Coiffard et al., 2020).

However, Spanish guidelines in 2018 recommend extending PAP to 10–15 days for LuTx recipients if donor cultures are positive for *P. aeruginosa* or the recipient has infectious lung disease due to the same pathogen. AST guidelines acknowledge the absence of definitive data on optimal PAP duration in LuTx recipients but note that some centers use at least 7 days postoperatively. They recommend involving infectious disease specialists for individualized decisions (Aguado et al., 2018). Even in the most recent update of GESITRA-IC/SEIMC, CIBERINFEC, and SET recommendations in 2025, no new indications on PAP duration are provided and further research are suggested to establish definitive guidelines (Rodríguez-Goncer et al., 2025).

Pascale et al. (2024) conducted a single-center retrospective study on PAP regimens to prevent early postoperative infections (EPOIs) in LuTx. They observed no significant difference in EPOI rates between single-agent and combination regimens. Furthermore, PAP durations were frequently extended unnecessarily, even when donor/recipient cultures were negative, with a median duration of 10 days. This extended prophylaxis did not reduce EPOI incidence, leading the authors to advocate for shorter PAP durations.

Importantly, only the Spanish guidelines consider a potential role for aerosolized antibiotics. They suggest inhaled therapy for LuTx recipients with respiratory tract colonization by ESBL-producing Enterobacterales, CRE, or when receiving a colonized graft.

Again, these recommendations support the use of nebulized antibiotics prior to transplantation in lung transplant eligible recipients with chronic P. aeruginosa infection, regardless of the pathogen’s antimicrobial resistance profile. In this context, administering nebulized colistin immediately after transplantation may help protect the bronchial anastomosis from infectious complications and reduce the risk of CLAD, particularly if P. aeruginosa continues to be isolated from respiratory secretions.

Recommended agents include aminoglycosides or colistin, guided by susceptibility testing, and delivered via appropriate nebulization devices, such as electronic or vibrating mesh nebulizers (Aguado et al., 2018).

However, further suggestions on PAP duration may derive from the Milano algorithm, which aims to profit by PCR’s rapid turnaround time and sensitivity to avoid ineffective treatments and safely shorten prophylaxis duration to five days post-surgery if no pathogens are detected (Lombardi et al., 2025b).

Table 1 shows a synoptic overview of the key recommendations from the latest available guidelines.

**Table 1:** Comparison of major guidelines on preoperative antibiotic prophylaxis for patients colonized with MDR-GNB undergoing solid organ transplantation (LuTx when specified).

Colonizing pathogen	SET/GESITRA-SEIMC/REIPI 2017		ESCMID/EUCIC 2022	
	Recommendation	Strength, level of evidence	Recommendation	Strength, level of evidence
Extended-spectrum cephalosporin-resistant Enterobacterales (ESCR-E)	Targeted prophylaxis ***Adjunctive considerations*** Avoid carbapenems if possible. Consider inhaled antibiotics for LuT recipients with respiratory tract colonization or receiving a colonized graft.	B, III B, III	Targeted prophylaxis ***Adjunctive considerations*** limit carbapenem use if alternatives available.	Ungraded - good practice statement, Expert opinion
Carbapenem Resistant Enterobacterales (CRE)	Standard prophylaxis ***Adjunctive considerations*** Targeted prophylaxis in high-endemicity context of CRE-SSIs. Consider inhaled antibiotics for LuTx recipients with respiratory tract colonization	B, III C, II C, II	Insufficient evidence to suggest for or against targeted prophylaxis	No recommendation
Carbapenem Resistant *Acinetobacter baumannii* (CRAB)	Standard prophylaxis	A, II	Insufficient evidence to suggest for or against targeted prophylaxis	No recommendation
MDR *P.aeruginosa*	Target prophylaxis for recipients with a septic lung disease	C, III	Not specifically addressed	Not specifically addressed
Duration of prophylaxis in patients colonized with MDR-GNB before surgery	3–5 days If respiratory cultures from both donor and recipient at the time of transplantation are reported as STERILE 10–15 days If respiratory cultures from both donor and recipient at the time of transplantation are reported as POSITIVE or the recipient has a septic lung disease	C, III	48–72 h	Ungraded - good practice statement, Expert opinion

MDR-GNB, multi-drug resistant Gram-negative bacteria; LuTx, lung transplantation; SSIs, surgical-site infections.

#### 4.2 Therapy of MDR-GNB infections in LuTx recipients

Recommendations for antimicrobial therapy of MDROs infections in SOT recipients do not differ from those for non-transplant patients, either in terms of agent/regimen selection or treatment duration. However, in LuTx recipients where the most frequent infectious complications involve the allograft, a significant challenge lies in achieving and maintaining appropriate antibiotic concentrations in the pulmonary district to ensure effective MICs.

Respiratory pathogens are typically categorized as extracellular or intracellular, targeting, respectively, the pulmonary epithelial lining fluid (ELF) and alveolar macrophages. These compartments have thus been recognized as critical sites for achieving therapeutic drug levels. Since 2011, Rodvold et al. (2011) have sought to clarify the distribution patterns of antibiotics in the ELF, analyzing over 80 studies that reported ELF concentrations and extracellular penetration ratios of various antibacterial agents. Their findings revealed considerable variability among drug classes. For example, aminoglycosides consistently reached the ELF, though at concentrations lower than in plasma. For beta-lactam agents, however, penetration was difficult to predict, even after accounting for factors like protein binding and molecular size. Conversely, macrolides, ketolides, fluoroquinolones, and linezolid demonstrated ELF/plasma concentration ratios >1.

This means that the antibiotics typically used to treat MDR-GNB infections may face significant limitations in attaining therapeutic concentrations in the lung, a challenge that exists even in healthy individuals but is exacerbated in LuTx recipients. Postoperative complications such as hemodynamic instability, bleeding, hypovolemia, sepsis, or pump failure can compromise drug delivery. Furthermore, acute kidney injury (AKI)—whether due to surgical complications, nephrotoxic immunosuppressants, antibiotics, or other medications—may necessitate renal replacement therapy (RRT), further complicating drug pharmacokinetics (Di Nardo et al., 2022).

Rando et al. (2024) explored these pharmacokinetic (PK) complexities in the context of newer beta-lactams (BLs) and beta-lactam/beta-lactamase inhibitor (BL/BLI) combinations developed to combat MDR GNB like cefiderocol, ceftolozane/tazobactam, ceftazidime/avibactam, imipenem/cilastatin/relebactam, and meropenem/vaborbactam. They concluded that due to the limited availability of ELF-specific PK data, there is no certainty that standard dosing regimens for these agents ensure adequate ELF penetration or efficacy in pneumonia. Most available studies inferred ELF exposure indirectly from plasma pharmacokinetics and probability of target attainment (PTA) models, despite the known variability of antibiotic distribution in pulmonary tissue, especially under critical illness. A low AUCELF/AUCplasma ratio might necessitate higher dosing to achieve therapeutic exposure in the lung, raising the associated risk of toxicity.

They also highlighted an important limitation in current research regarding the frequent exclusion of patients undergoing RRT or extracorporeal membrane oxygenation (ECMO), even though both interventions are known to significantly alter beta-lactam pharmacokinetics. These changes are often further influenced by the critical condition of the patients and the presence of multiple organ failure, especially in ventilated cases of hospital-acquired or ventilator-associated pneumonia (Roberts and Lipman, 2009).

About this aspect, Taccone et al. (2021) described how β-lactam blood concentrations are often below the desired target to treat less susceptible Gram-negative strains in early phase after LuTx. They demonstrated how “insufficient” drug concentrations were associated with a higher proportion of early colonization with MDR strains and/or early infections. In fact, they suggested the need for future studies to evaluate early antibiotic drug concentrations and to define optimal prophylactic regimens.

In conclusion, further pharmacokinetic studies are urgently needed to guide appropriate dosing of BL and BL/BLI antibiotics in critically ill patients, particularly those with MDR infections and on RRT or ECMO. For these complex scenarios, broader implementation of therapeutic drug monitoring (TDM)-driven approaches appears warranted, especially where significant PK/PD deviations are expected, an approach supported by recent studies (Gatti et al., 2021, 2023).

##### 4.2.1 Systemic antibiotics

The first relevant guidelines addressing MDR-GNB infections in SOT recipients were released in 2019 by the American Society of Transplantation Infectious Diseases Community of Practice. However, as newer antimicrobials such as imipenem-relebactam, cefiderocol, and eravacycline were not yet widely available at the time, more recent guidelines are below discussed in greater detail (Pouch and Patel, 2019).

In 2021, ESCMID published guidelines for the treatment of infections caused by MDR-GNB, including insights relevant to severe infections such as early post-lung transplantation pneumonia supposed to be (Paul et al., 2022).

For infections due to third-generation cephalosporin-resistant Enterobacterales (3GCephRE), ESCMID strongly recommends carbapenems (imipenem or meropenem) as targeted therapy. For CRE, ceftazidime/avibactam or meropenem-vaborbactam are conditionally recommended if the pathogen is susceptible. In cases of CRE producing metallo-*β*-lactamases or resistant to all other available agents, cefiderocol or the combination of ceftazidime/avibactam plus aztreonam is suggested. Notably, all these agents are recommended as monotherapy.

In severe infections caused by difficult-to-treat carbapenem-resistant *P. aeruginosa* (CRPA), ceftolozane-tazobactam is strongly recommended if active *in vitro*, while the guidelines do not make a recommendation for or against combination therapy due to insufficient evidence.

For CRAB causing hospital- or ventilator-associated pneumonia (HAP/VAP), high dose ampicillin/sulbactam is conditionally recommended when the strain is susceptible. In cases of sulbactam resistance, treatment options include a polymyxin or high dose tigecycline. Combination regimens using at least two in vitro-active agents—such as aminoglycosides, tigecycline, or extended-infusion high-dose meropenem (if MIC ≤ 8 μg/mL)—are also advocated. A conditional recommendation against the use of cefiderocol in CRAB infections is made based on low-level evidence.

For pan-resistant carbapenem-resistant Gram-negative bacteria (CR-GNB), including those resistant to polymyxins, the use of the least resistant agent(s), selected according to MICs relative to susceptibility breakpoints, is considered good clinical practice (Paul et al., 2022).

In 2024, the Infectious Diseases Society of America (IDSA) updated its guidelines on the treatment of MDR-GNB infections (Tamma, 2024). Carbapenems are recommended as treatment for moderate to severe infections (excluding urinary tract infections) caused by ESBL-producing Enterobacterales, with the possibility of switching to oral agents such as trimethoprim-sulfamethoxazole (TMP-SMX), ciprofloxacin, or levofloxacin when clinical improvement and susceptibility allow. For organisms with inducible AmpC β-lactamase production (e.g., *E. cloacae complex, K. aerogenes, C. freundii*), cefepime may be considered.

For CRE, IDSA offers specific guidance based on the type of carbapenemase produced. For KPC-producing Enterobacterales, preferred agents include meropenem-vaborbactam, ceftazidime-avibactam, and imipenem-cilastatin-relebactam, while cefiderocol may be used as an alternative. For OXA-48-like producers, ceftazidime-avibactam is preferred, with cefiderocol again as an alternative. For metallo-β-lactamase producers, the same treatment options as ESCMID are supported. All these therapies are recommended as monotherapy when the β-lactam agent shows in vitro activity.

If β-lactam antibiotics are not viable options due to allergy or intolerance, specifically in non-urinary tract CRE infections, tigecycline and eravacycline are proposed as alternatives.

Unlike ESCMID, the IDSA guidelines suggest a broader range of options for infections caused by difficult-to-treat *P. aeruginosa* (DTR-PA), including not only ceftolozane/tazobactam but also ceftazidime-avibactam, imipenem-cilastatin-relebactam, and cefiderocol, provided susceptibility is confirmed. Again, monotherapy is preferred when effective.

For CRAB infections, IDSA recommends combination therapy including a sulbactam-based agent. Sulbactam-durlobactam, in combination with a carbapenem (imipenem-cilastatin or meropenem), is the preferred option. Where sulbactam-durlobactam is unavailable, high-dose ampicillin-sulbactam in combination with one or more agents—such as polymyxin B, minocycline, tigecycline, or cefiderocol—should be considered. GESITRA-IC/SEIMC, CIBERINFEC, and SET most recent recommendations, in cases where sulbactam-durlobactam is unavailable, suggest an alternative regimen including high-dose ampicillin-sulbactam in combination with cefiderocol, imipenem, meropenem, IV minocycline, tigecycline or eravacycline. In stable patients with non-severe infections, sulbactam-durlobactam monotherapy could be used in monotherapy if available (Rodríguez-Goncer et al., 2025).

Notably, the IDSA guidelines also address treatment options for *S. maltophilia* infections, recommending two agents among cefiderocol, minocycline, TMP-SMX, or levofloxacin, as well as the combination of ceftazidime-avibactam and aztreonam (Tamma, 2024).

A fourth set of relevant recommendations comes from a 2022 consensus document by Tiseo et al. (2022), endorsed by multiple Italian societies (SIMIT, SITA, GISA, AMCLI, SIM).

These guidelines generally align with ESCMID and IDSA recommendations, offering a comprehensive overview of the management of MDR bacterial infections.

Table 2 presents a comparative summary of key recommendations from these four expert panels for the treatment of MDR-GNB infections, reporting HAP/VAP-specific guidance when provided, and otherwise referring to recommendations for severe infections.

**Table 2:** Comparison of major guidelines on infections due to MDR-GNB (only AST specifically addressed to patients undergoing solid organ transplantation).

Pathogen	AST 2019	ESCMID 2021	SIMIT/SITA/GISA/AMCLI/SIM 2022	IDSA 2024
Extended-spectrum cephalosporin-resistant Enterobacterales (ESCR-E)	Carbapenems	Carbapenems (Imipenem or Meropenem) ***Adjunctive considerations*** Stepdown targeted therapy once patients are stabilized Avoid new BL/BLI	Note addressed	Carbapenems (Imipenem or Meropenem) ***Adjunctive considerations*** Stepdown targeted therapy once patients are stabilized Cefepime if risk of AmpC production (i.e, *E. cloacae complex, K. aerogenes, and C. freundii*).
Carbapenem Resistant Enterobacterales (CRE)	CAZ/AVI or MER/VAB *MBLs producing strains* CAZ/AVI + ATM	MER/VAB or CAZ/AVI *MBLs producing strains* CFDC or CAZ/AVI + ATM ***Adjunctive considerations*** Combination therapy is NOT recommended	*KPC producing strains* CAZ/AVI or MER/VAB Alternatives: IMI/REL or CFDC *OXA-48 producing strains* CAZ/AVI *MBLs producing strains* CAZ/AVI + ATM Alternatives: CFDC	K*PC producing strains* CAZ/AVI or MER/VAB or IMI/REL Alternatives: CFDC *OXA-48 producing strains* CAZ/AVI *MBLs producing strains* CAZ/AVI + ATM or CFDC ***Adjunctive considerations*** Combination therapy is NOT recommended
MDR *P. aeruginosa*	TOL/TAZ or CAZ/AVI ***Adjunctive considerations*** High-dose continuous or extended-infusion Adjunctive aerosolized colistin or tobramycin for pneumonia	TOL/TAZ	TOL/TAZ or CAZ/AVI	TOL/TAZ or CAZ/AVI or IMI/REL Alternatives: CFDC ***Adjunctive considerations*** Combination therapy is NOT suggested CFDC if MBLs production is detected Nebulized antibiotics for the treatment of respiratory infections are NOT suggested
Carbapenem Resistant *Acinetobacter baumannii* (CRAB)	Carbapenem + colistin or polymyxin B Alternatives: AMP/SUL or MIN	HD AMP/SUL + polymyxin or aminoglycoside or HD TYG ***Adjunctive considerations*** CFDC NOT recommended	Infectious diseases specialist consultation ***Adjunctive considerations*** Consider CFDC	SUL/DUR + Imipenem or Meropenem Alternatives: HD AMP/SUL + polymyxin B or MIN or TYG or CFDC
*Stenotrophomonas maltophilia*	HD TMP/SMX			CAZ/AVI + ATM or Two among CFDC. MIN, TMP/SMX, LFX
Comments	Pursue source control Early transplant infectious disease consultation	For pan-resistant CR-GNB (resistant also to polymyxins), treatment with the least resistant antibiotic/s		

MDR-GNB, multi-drug resistant Gram-negative bacteria; HAP/VAP, hospital-acquired pneumonia/ventilator-associated pneumonia; CAZ/AVI, ceftazidime/avibactam; MER/VAB, meropenem/vaborbactam; ATM, aztreonam; TOL/TAZ, ceftolozane/tazobactam; AMP/SUL, ampicillin/sulbactam; MIN, minocicline, HD, high dose; TMP/SMX, trimethoprim/sulfamethoxazole; BL/BLI, β-lactam/β-lactamase inhibitor; CFDC, cefiderocol; TYG, tygecicline, CR-GNB, carbapenem-resistant Gram-negative bacteria; IMI/REL, imipenem/relebactam. If specified, recommendation for HAP/VAP or sever infection are reported.

##### 4.2.2 Nebulized antibiotics

The difficulty many antimicrobials face in achieving optimal PK/PD targets in the treatment of pneumonia is one of the main reasons why inhaled antibiotics represent an attractive option for managing respiratory tract infections caused by MDR-GNB. They allow effective drug delivery directly to the site of infection while limiting systemic exposure and toxicity (Wood, 2011).

Although most evidence for inhaled antibiotics comes from patients with VAP or cystic fibrosis, their use has also been described in treating respiratory colonization by ESBL-producing Enterobacteriaceae in lung transplant recipients (Aguilar-Guisado et al., 2007).

Beyond the already discussed use of inhaled antibiotics in colonized patients, various studies have explored their role in treating MDR-GNB pneumonia.

In 2017, following a systematic review, ESCMID released a position paper on the use of nebulized antibiotics in invasively mechanically ventilated adults (Rello et al., 2017).

Regarding MDR-GNB, ESCMID recommends against using nebulized aminoglycosides or colistin. Although a meta-analysis of observational studies evaluating adjunctive nebulized therapy for VAP caused by resistant pathogens showed improved clinical resolution, shorter duration of mechanical ventilation, and lower VAP-related mortality, the overall quality of evidence was deemed very low. Moreover, the safety analysis revealed a higher incidence of respiratory complications, particularly in patients with severe hypoxemia (PaO₂/FiO₂ < 200) or poor pulmonary reserve due to their propensity for rapid lung de-recruitment. No significant differences were found in systemic toxicity (e.g., nephrotoxicity or neurotoxicity). However, concerns remain, as nebulized aminoglycosides can enter the systemic circulation and reach toxic levels when co-administered intravenously. A similar increase in renal toxicity has been observed with colistin when administered both intravenously and via nebulization. Furthermore, the potential impact of nebulized therapy on airway microbiota remains unclear.

Consistent with this position, the 2024 IDSA guidelines also recommend against the use of nebulized antibiotics as adjunctive therapy for pneumonia caused by DTR-PA or CRAB, citing the lack of observed benefit in clinical trials, concerns over uneven drug distribution in infected lungs, and risks of respiratory complications such as bronchospasm (Tamma, 2024).

In contrast, the 2019 AST guidelines on MDR-GNB infections in SOT recipients provide a weak recommendation in favor of adjunctive inhaled colistin or tobramycin for the treatment of pseudomonal pneumonia and support the use of inhaled colistin as an adjunct to systemic therapy or as monotherapy in nonbacteremic respiratory infections caused by CRAB (Pouch and Patel, 2019).

### 4.3 Unusual MDROs: original cases

First scientific evidences reporting a greater prevalence of gram negative bacteria compared to gram positive ones as responsible for post-transplant pneumonia, occurs in the early 2000s; as Aguilar-Guisado reported among Gram-negative pathogens, *P. aeruginosa* was the leading cause of hospital-acquired pneumonia (HAP) after LuTx, accounting for up to 25% of cases (Aguilar-Guisado et al., 2007).

Additionally, *K. pneumoniae* is often the most commonly identified extensively drug-resistant (XDR) pathogen, reported in up to one-third of LuTx recipients in a Turkish cohort of SOT recipients, with a 7-day mortality rate of approximately 36% (Yanik Yalçin et al., 2021).

Beyond these more frequently encountered bacteria, rare or atypical Gram-negative pathogens have also been implicated in infectious complications post-LuTx. As summarized by Farfour et al. (2023), these infections pose significant clinical challenges due to several factors:

- under-recognition due to their rarity,
- difficulties in laboratory detection and identification,
- frequent multidrug resistance and lack of standardized susceptibility guidelines,
- challenges in distinguishing colonization from true infection, and.
- unclear risk and control measures for nosocomial cross-transmission.

Their narrative review highlighted NFGNB isolated from 17 LuTx recipients including organisms from the genera *Acetobacter, Bordetella, Chryseobacterium, Elizabethkingia, Inquilinus*, and *Pandoraea*. These organisms are generally regarded as environmental saprophytes or opportunists (except for *Bordetella*) but may exhibit pathogenic behavior in immunocompromised hosts (Farfour et al., 2023). In retransplant patients, Gram-negative organisms predominated throughout follow-up, with *Elizabethkingia* being the dominant early isolate, representing 31.6% of BAL cultures within the first month and remaining a major pathogen for up to 6 months post-transplantation (Han et al., 2025).

Understanding their clinical significance often requires a combination of clinical, radiologic, and biochemical assessment. When isolates are available, antimicrobial susceptibility testing is often problematic: EUCAST and CLSI lack standardized breakpoints or incubation guidelines (e.g., inoculum size, temperature, duration) for many of these species. Thus, susceptibility results are often extrapolated from unrelated organisms such as *P. aeruginosa* or *Burkholderia* spp. (Sfeir, 2020).

Data on resistance mechanisms are sparse and mainly derived from case reports or small case series, often in cystic fibrosis patients with extensive prior antibiotic exposure (Farfour et al., 2023).

*In vitro* studies show that many of these pathogens are intrinsically resistant to beta-lactams and beta-lactam/beta-lactamase inhibitor combinations, with occasional carbapenem susceptibility. For example, *Elizabethkingia* spp. produces chromosomally encoded metallo-beta-lactamases (Hu et al., 2020).

Other resistance mechanisms include efflux pumps, target mutations, and methylation, contributing to resistance against beta-lactams and other antibiotic classes such as fluoroquinolones (Coward et al., 2020; Lim et al., 2016; Pino et al., 2025)

Even rare pathogens such as *Bordetella hinzii* have been implicated in post-LuTx pneumonia. This organism exhibits intrinsic resistance via broad-spectrum beta-lactamase production and may develop further resistance under targeted treatment (Vulturar et al., 2024).

However, the true pathogenic potential of *B. hinzii* remains uncertain due to the limited number of reported clinical cases (Maison-Fomotar and Sivasubramanian, 2021).

As such, Vulturar et al. (2024) emphasized the need for multicenter collaborative studies to better understand the prevalence, risk factors, and outcomes associated with these infections.

In recent years, *A. xylosoxidans* and *S. maltophilia* have attracted increasing attention due to their potential to develop MDR and pan-resistant phenotypes. In a study by Lobo et al. (2015), LuTx recipients colonized or infected with these organisms before transplantation (regardless of resistance profile) showed no difference in overall post-transplant survival. However, a higher incidence of BOS-related mortality was observed in the *Achromobacter* spp. group, suggesting possible non-infectious immune dysregulation triggered by this pathogen (Lobo et al., 2015).

*S. maltophilia* is known for its arsenal of resistance determinants, including L1 metallo-beta-lactamases and L2 serine-beta-lactamases, rendering most beta-lactams and their combinations ineffective (Chang et al., 2015; Mojica et al., 2019).

A recent retrospective study on LuTx recipients by Pilmis et al. (2024) found that 6% of initial *S. maltophilia* isolates were multidrug resistant. Notably, microbiological recurrence was associated with acquired resistance in 68% of cases.

*S. maltophilia* is an environmental organism capable of forming biofilms and surviving on hospital equipment, posing serious risks to immunocompromised patients (Brooke, 2012).

The clinical significance of isolating environmental, yet potentially opportunistic, pathogens from the upper airways of LuTx recipients remains uncertain. In particular, it is still unclear when, or whether, targeted antibiotic therapy should be initiated in asymptomatic patients. To explore this issue, Hofmann et al. (2016) conducted a small retrospective study involving asymptomatic LuTx recipients colonized with *S. maltophilia* in the upper airways. By comparing patients who received antibiotic treatment to those who did not, the authors observed no significant differences in lung function or systemic inflammatory markers. Based on these findings, they concluded that the isolation of *S. maltophilia* in asymptomatic LuTx recipients should not be considered clinically relevant in the short term.

## 5 Conclusion

In recent years, no absolute contraindications to lung transplantation have been established regarding the presence of MDR-GNB infections in either donors or recipients. However, the well-documented negative impact of these infections on both patient and graft survival, combined with the limited availability of effective antimicrobials, highlights the urgent need to optimize prevention and management strategies in LuTx recipients.

A deeper understanding of the relationship between colonization and infection is essential, and the implementation of rapid diagnostic tools should be prioritized to guide timely and targeted antimicrobial therapy—both in deciding whether treatment is warranted and in selecting the most appropriate agents. In this field, metagenomic next-generation sequencing (mNGS) could be of potential value. As Ju et al. (2022) demonstrated, compared with conventional detection methods, mNGS is associated with a higher capacity of identifying infection and could help differentiate infectious diseases in LuTx recipients from non-infectious ones. Maybe, interesting data will derive from the Milano Algorithm from Lombardi et al. (2025b).

Further research is also needed to assess whether current antibiotic regimens are appropriately used in critically ill patients undergoing renal replacement therapy or extracorporeal membrane oxygenation—frequent interventions in the postoperative care of LuTx recipients. Similarly, the therapeutic role of inhaled antibiotics remains to be clearly defined. Bacteriophage therapy or monoclonal antibodies, for example, could be helpful news in this field of study. Above all, phages are a novel and highly targeted tool that may be applied to the MDR-GNB derived infections in LuTx setting. As an example, an ongoing international registry of patients with CF and LuTx recipients colonized with *Burkholderia cenocepacia* complex organisms is collecting bacterial isolates from the enrolled patients and using these isolates to develop a lytic phage library for eventual use in a pilot clinical trial (UC San Diego School of Medicine, 2023). Alternatively, phage could be deployed in patients on the waitlist as an effort to eradicate infection before transplantation. There is also a potential for targeted phage therapy to optimize the post-transplant respiratory microbiome and affect not just infectious complications but also long-term graft function and survival (Aslam, 2023).

Addressing these gaps in knowledge is crucial to improving outcomes and ensuring the best possible survival for patients undergoing the only definitive treatment option for end-stage lung diseases.
